# Extracellular calcium triggers unique transcriptional programs and modulates staurosporine-induced cell death in *Neurospora crassa*

**DOI:** 10.15698/mic2014.09.165

**Published:** 2014-08-09

**Authors:** A. P. Gonçalves, João Monteiro, Chiara Lucchi, David J. Kowbel, J. M. Cordeiro, Paulo Correia-de-Sá, Daniel J. Rigden, N. L. Glass, Arnaldo Videira

**Affiliations:** 1 ICBAS-Instituto de Ciências Biomédicas de Abel Salazar, Universidade do Porto, Rua de Jorge Viterbo Ferreira 228, 4050-313 Porto, Portugal.; 2 IBMC-Instituto de Biologia Molecular e Celular - Universidade do Porto, Rua do Campo Alegre 823, 4150-180 Porto, Portugal.; 3 Plant and Microbial Biology Department, The University of California, Berkeley, CA 94720, USA.; 4 UMIB-Unidade Multidisciplinar de Investigação Biomédica, Universidade do Porto, Rua de Jorge Viterbo Ferreira 228, 4050-313 Porto, Portugal.; 5 Institute of Integrative Biology, University of Liverpool, Liverpool, L69 7ZB, United Kingdom.

**Keywords:** calcium, cell death, ROS, Ca2+-binding motif, Neurospora crassa

## Abstract

Alterations in the intracellular levels of calcium are a common response to cell death stimuli in animals and fungi and, particularly, in the *Neurospora crassa* response to staurosporine. We highlight the importance of the extracellular availability of Ca^2+^ for this response. Limitation of the ion in the culture medium further sensitizes cells to the drug and results in increased accumulation of reactive oxygen species (ROS). Conversely, an approximately 30-fold excess of external Ca^2+^ leads to increased drug tolerance and lower ROS generation. In line with this, distinct staurosporine-induced cytosolic Ca^2+^ signaling profiles were observed in the absence or presence of excessive external Ca^2+^. High-throughput RNA sequencing revealed that different concentrations of extracellular Ca^2+^ define distinct transcriptional programs. Our transcriptional profiling also pointed to two putative novel Ca^2+^-binding proteins, encoded by the NCU08524 and NCU06607 genes, and provides a reference dataset for future investigations on the role of Ca^2+^ in fungal biology.

## INTRODUCTION

Calcium (Ca^2+^) is crucial for diverse processes in fungi, including tip growth, virulence, circadian rhythms, nutrient sensing, cell wall regeneration, chemotropic interaction and cell death 1. Ca^2+^ and cell death are intimately connected, especially because of the vital role of Ca^2+^ as an intracellular messenger affecting numerous signaling pathways that determine cell fate. Therefore, it is not surprising that cells commonly respond to cell death stimuli with rises in the intracellular levels of this divalent ion. On one hand, these transient or stable modifications in the levels of Ca^2+^ can operate as survival signals, leading to the activity of anti-death proteins or stimulating transcriptional defence responses. On the other hand, the loss of Ca^2+^ homeostasis can behave as a pro-death signal, since in some systems overload or a simple perturbation of the distribution of the ion within storage organelles is sufficient to trigger cell death 2. In yeast species like *Saccharomyces cerevisiae*, *Candida albicans* and *Cryptococcus neoformans*, cytosolic Ca^2+^ increases are a common feature of the cellular response to a number of antifungal stresses, including pheromone 3, the plant essential oils carvacrol 4 and eugenol 5, amiodarone 3,6, and ER stress-inducing agents like tunicamycin and azole drugs 7,8. In *N. crassa*, a rise in the cytosolic levels of Ca^2+^ was associated with cell death induced by chitosan 9, *Penicillium chrysogenum* protein PAF 10, PAF26 11 and staurosporine 12.

We have been using *N. crassa *to investigate the molecular mechanisms underlying the cell death process induced by the bacterial alkaloid staurosporine. Treatment with staurosporine leads to a complex response that includes the efflux of reduced glutathione (GSH), with a concomitant change in the intracellular redox state to a more oxidative environment and the consequent accumulation of reactive oxygen species (ROS) 13,14. The drug also activates a drug resistance pathway that comprises the zinc binuclear cluster transcription factor CZT-1 15 and ABC-3, an ATP-binding cassette (ABC) transporter 16. A combined genetic and pharmacological strategy coupled to measurements of cytosolic alterations in Ca^2+^ levels with the photoreporter aequorin revealed a three-step signature response to the drug that involves mobilization of the ion from intracellular stores as well as uptake from the external medium. *N. crassa* is a good model for the study of Ca^2+^ dynamics as sequencing of its genome revealed a rich and versatile assortment of molecules involved in Ca^2+^ homeostasis, including channels, pumps and signaling transducers 17,18,19. A substantial fraction of the genome, however, remains to be characterized and additional unknown members of Ca^2+^ machinery may exist. The best-studied Ca^2+^-binding moiety is the classical EF-hand, in which a Ca^2+^-interacting loop is flanked by two helices 20. However, there are variations on this motif, especially at the structural level, and it is now accepted that more than a dozen different types of Ca^2+^-binding domains exist 21. In particular, several proteins classes were shown to carry convergently evolved Ca^2+^-binding Dx[DN]xDG motifs. The distinct evolutionary origins of the motif are evident from the variability of structural contexts flanking the Ca^2+^-binding region 22,23. Though initially identified in bacteria 24, it was later perceived that this motif is widespread across all kingdoms 22,23, including in a fungal caleosin 23,25 and lectin from *Psathyrella velutina *22,26.

We have recently shown that staurosporine-induced cell death in *N. crassa* involves a precise sequence of Ca^2+^ signaling events which are completely abolished by the inhibition of extracellular Ca^2+^ uptake 12. Thus, we investigated the effects of the drug in *N. crassa* cells growing in limited or excessive amounts of the ion. The results presented here revealed that extracellular Ca^2+ ^modulates cell death and the transcriptional alterations induced by staurosporine, and led to the identification of two novel putative Ca^2+^-binding proteins, encoded by the NCU08524 and NCU06607 genes.

## RESULTS

### The availability of extracellular Ca^2+^ impacts staurosporine-induced cell death and intracellular Ca^2+^ signaling

**Figure 1:**
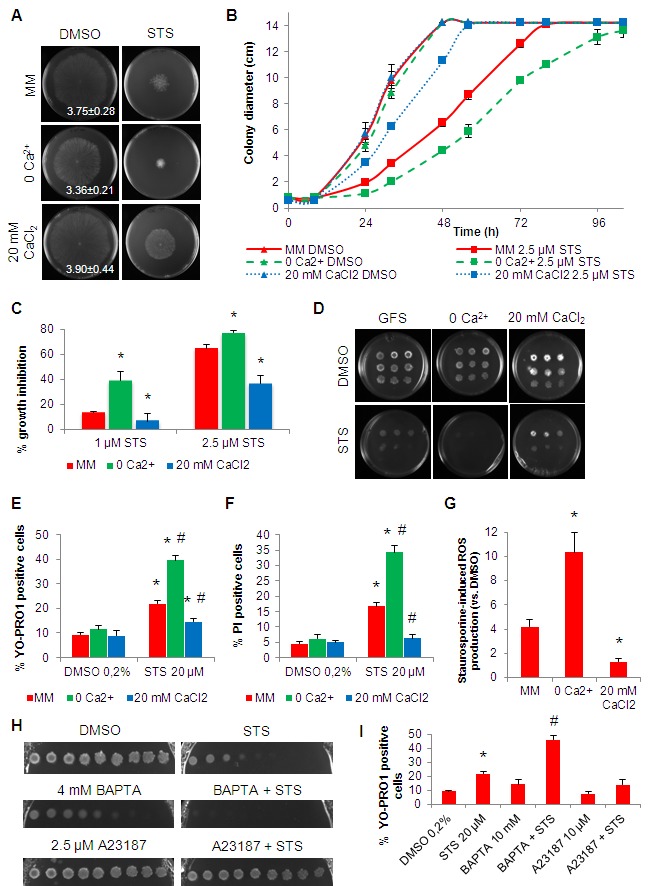
FIGURE 1: Extracellular Ca^2+^ modulates the *N. crassa *sensitivity to staurosporine. **(A-C)** Conidia were inoculated on the centre of a Petri dish containing Vogel’s MM with 2.5 μM staurosporine (STS) and different concentrations of Ca^2+^. Growth at 32h **(A)**, over time **(B)** and the percentage of growth in STS-treated cells versus control **(C)** are shown. Growth rates (mm/h) are indicated in the left panels in **(A)**. *, p-value <= 0.05. **(D)** Serial dilutions (from left to right) of conidia spotted in GFS agar medium supplemented with 5 μM STS and different concentrations of Ca^2+^ were incubated for 3 days. **(E-F)** Cell death following treatment of the cells with 20 μM staurosporine in the indicated liquid culture media was evaluated by flow cytometry quantification of positive cells for YOPRO-1 **(E)** or PI **(F)**. *, p-value <= 0.05 for the comparison STS versus DMSO in each media; #, p-value <= 0.05 for the comparison between media after the treatment with STS. **(G)** The fold increase in cellular ROS accumulation following treatment with 20 μM staurosporine in the indicated liquid culture media was evaluated by staining with DHR123. *, p-value <= 0.05. **(H-I)** Growth inhibition and cell death in 2.5 μM STS-treated cells in the presence of BAPTA or A23187 was assessed by spots in GFS medium **(H)** or cell growth in liquid medium followed by staining with YOPRO-1 **(I)** *, p-value <= 0.05 for the comparison STS versus DMSO; #, p-value <= 0.05 for the comparison between STS alone and BAPTA+STS.

Intracellular Ca^2+^ dynamics play an important role during staurosporine-induced cell death in *N. crassa*, eliciting a complex response that includes Ca^2+^ release from internal stores as well as uptake from the extracellular space 12. Thus, we tested the response to staurosporine of cells growing with different Ca^2+^ concentrations. We prepared a culture medium, designated ‘no Ca^2+^’, by removing CaCl_2_ from Vogel’s minimal medium (MM) 27. Small amounts of Ca^2+^ as impurities from the other reagents in the solution allowed *N. crassa* to grow well under such conditions (Fig. 1A, middle panel). In contrasting experiments, we supplemented medium with 20 mM CaCl_2_ (’20 mM CaCl_2_’ medium), which represents an approximately 30 fold (but not toxic) increase as compared with the 0.68 mM CaCl_2_ present in standard Vogel’s medium (Fig. 1A, lower panel). We inoculated wild type cells on the centre of Petri dishes containing standard, no Ca^2+^ or 20 mM CaCl_2_ solid Vogel’s MM supplemented with staurosporine and measured radial growth during a 104 hour time course. The drug inhibited growth in all media (Fig. 1A-C). However, the staurosporine inhibitory effect was amplified in the absence of Ca^2+^ (e.g., ~39% of inhibition after 32 hours of treatment with 1 μM staurosporine in no Ca^2+^ versus ~14% in standard MM). On the other hand, growth inhibition was partially overcome in 20 mM CaCl_2_ medium (e.g., ~37% of inhibition after 32 hours of treatment with 2.5 μM staurosporine in 20 mM CaCl_2_ versus ~65% in standard MM). In GFS medium, which contains sorbose that promotes colonial growth 28, the outcome of modifying the extracellular concentration of Ca^2+^ was similar: the effects of staurosporine were exacerbated in no Ca^2+^ medium whereas growth inhibition was partially suppressed in the presence of 20 mM CaCl_2_, (Fig. 1D). Enhancement and suppression of inhibition of growth by absence of Ca^2+^ and 20 mM CaCl_2_, respectively, was not reproduced when cells are treated with phytosphingosine (data not shown), another cell death inducer in *N. crassa*, supporting previous observations that staurosporine and phytosphingosine act by distinct mechanisms 14,29,30.

In order to check cell death, we analysed cell staining with YOPRO-1 (early apoptosis marker) or PI (late apoptosis/necrosis marker) by flow cytometry. A 2-hour treatment with staurosporine in standard MM resulted in ~22% and ~17% of YOPRO-1 and PI positive cells, respectively (Fig. 1E-F). In no Ca^2+^ medium, these levels were augmented to ~40% (p-value ~ 0) and ~34% (p-value = 0.029), respectively (Fig. 1E-F). In contrast, the percentage of YOPRO-1 and PI-positive cells was reduced to ~15% (p-value = 0.042) and ~6% (p-value = 0.050), respectively, in 20 mM CaCl_2_ medium.

The production of ROS is an essential event during the response of *N. crassa* to staurosporine, as the addition of antioxidants blocks cell death 13. We asked if the availability of Ca^2+^ in the culture medium influenced cellular ROS production induced by staurosporine. While in standard MM cells stressed for 30 minutes with staurosporine displayed a ~4.2 fold-increase in ROS accumulation, the equivalent accumulation was ~10.4 (p-value = 0.001) and ~1.3 (p-value = 0.014) in no Ca^2+^ and 20 mM CaCl_2_ media, respectively (Fig. 1G). Given the importance of ROS formation for cell death provoked by staurosporine, this Ca^2+^-ROS dependence is likely associated with the distinct cell death phenotypes in the different culture media.

To further stress the modulatory effect of altering the levels of Ca^2+^ in staurosporine-induced cell death, we combined the drug with the Ca^2+^ chelator BAPTA and the Ca^2+^ ionophore A23187 (calcimycin) in GFS plates with standard MM. As expected, extracellular Ca^2+ ^ blockage with BAPTA synergized whereas elevation of intracellular Ca^2+^ with A23187 antagonized the effects of staurosporine (Fig. 1H). In agreement, pre-incubation with BAPTA significantly enhanced apoptotic levels induced by staurosporine, whereas they were partially inhibited by A23187, as measured by YOPRO-1 staining (Fig. 1I). Altogether, these data consistently indicate that the absence of Ca^2+^ enhances staurosporine-induced cell death, whereas excess (but not toxic) concentrations of Ca^2+^ partially prevent it.

**Figure 2:**
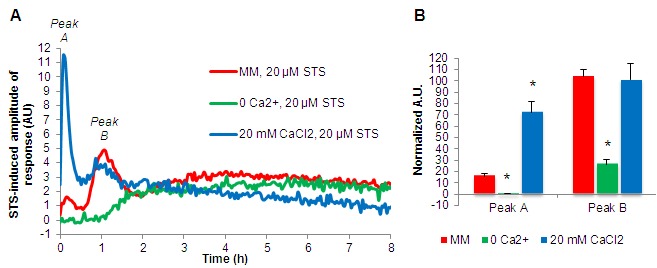
FIGURE 2: Extracellular Ca^2+^ availability affects intracellular Ca^2+^ dynamics in response to staurosporine. **(A)** Aequorin-expressing wild type cells were cultured for 6 hours in the indicated media and after the addition of 20 μM staurosporine (STS), luminescence was followed over time. The STS-induced amplitude of response is shown. **(B)** Quantification (in arbitrary units) of the cytosolic Ca^2+^ peaks "A" and "B" (indicated in A). *, p-value < 0.05.

Using cells expressing the cytosolic Ca^2+^ reporter gene aequorin, we described recently that staurosporine induces a three-step Ca^2+^ response comprising three main signals called "A", "B" and "C". Peak "A" occurs immediately after the addition of the drug and lasts approximately 20 minutes; peak "B" appears after 35-40 minutes and lasts approximately 80 minutes; phase "C" is a prolonged and continuous elevation of cytosolic Ca^2+^ 12. In the absence of extracellular Ca^2+^, the Ca^2+^ response to staurosporine was compromised, with peaks "A" and "B" showing substantial reduction (Fig. 2A-B). In medium with 20 mM CaCl_2_ there was a strong boost in peak "A", while peak "B" remained similar to standard MM. These alterations in intracellular Ca^2+^ dynamics are in agreement with our findings that extracellular Ca^2+^ uptake is important for the Ca^2+^ signaling response to staurosporine 12.

### Distinct transcriptional programs in staurosporine-treated cells depend on the extracellular Ca^2+^ availability

**Figure 3:**
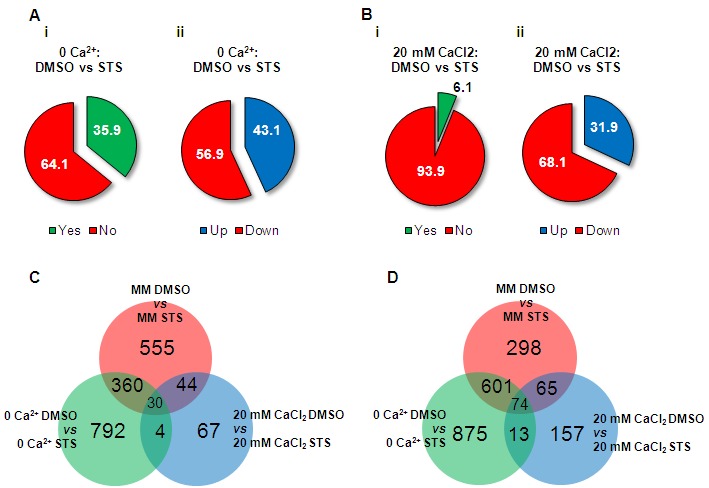
FIGURE 3: Overview of the *N. crassa* transcriptional response to staurosporine in minimal media containing different concentrations of Ca^2+^. **(A-B)** The percentages of genes with altered expression upon treatment with staurosporine (i) and the fraction of induced and repressed genes (ii) were calculated for culture medium with no Ca^2+^ **(A)** or with 20 mM CaCl_2_ **(B)**. **(C-D)** Venn diagrams were used to assess the amount of Ca^2+^-specific staurosporine-induced **(C)** and -repressed genes **(D)**. General statistics for Ca^2+^-specific transcriptional responses are included.

Treatment with 20 μM staurosporine led to significant alterations in the expression of ~28% of the *N. crassa *genes (1921 genes) 15. The extent of this transcriptional response was amplified in the absence of Ca^2+^ (~36% of genes (2749 genes) were altered by the drug), and markedly suppressed by 20 mM of CaCl_2_ (~6% genes (454 genes) showed altered profiles) (Fig. 3A-B). In standard medium, approximately half of the genes were induced and approximately half were repressed by staurosporine 15. In no Ca^2+^ medium, 43.1% of the altered genes were upregulated, whereas 56.9% where downregulated. Remarkably, in the presence of excessive Ca^2+^ most of the altered genes were repressed (~68%). The complete dataset obtained from these RNA-seq experiments is presented as File S1.

We analysed the distribution of the individual genes induced and repressed by staurosporine in the different culture media. In the absence of Ca^2+^, 792 (66.8%) of the induced genes were specific to this condition while in 20 mM CaCl_2_ this corresponded to 67 (46.2%) genes (Fig. 3C and File S1). Among repressed genes, 875 (56.0%) and 157 (50.8%) genes were specific to no Ca^2+^ and 20 mM CaCl_2_ media, respectively (Fig. 3D and File S1). Only minor fractions of the altered genes (30 and 74 genes, induced and repressed, respectively) were common to all conditions.

FunCat was used to examine the enrichment of functional categories in the different gene sets (File S2; Table S1-S2). The most interesting Ca^2+^ concentration-specific differences were found among the downregulated genes. In the absence of Ca^2+^, staurosporine led to the downregulation of genes included in numerous categories. These encompass various groups within the ‘Metabolism’ super-category such as ‘metabolism of the aspartate family’, ‘purine nucleotide/nucleoside/nucleobase metabolism’, ‘phosphate metabolism’ and ‘tetracyclic and pentacyclic triterpenes metabolism’. Also, there was a specific repression of genes involved in ‘Cell cycle and DNA processing’ and ‘Transcription’, namely ‘mRNA synthesis’ and ‘mRNA processing’. Categories related to stress responses like ‘Protein fate’, ‘unfolded protein response’, ‘cellular sensing and response to external stimulus’, ‘cell growth’, ‘anti-apoptosis’ were also repressed. Finally, while in standard MM there was a downregulation of ‘aerobic respiration’ genes, in no Ca^2+^ medium, other bioenergetic pathways were repressed: ‘glycolysis and gluconeogenesis’ and ‘pentose-phosphate pathway’. These data indicates that a highly complex transcriptional program is established by staurosporine-treated cells growing in the absence of Ca^2+^ resulting in the repression of several genes related with an anti-stress response. It seems that, when Ca^2+^ is limited, staurosporine causes the downregulation of cellular defences and this may be related to the high susceptibility of these cells to the drug.

### Limited and excess of Ca^2+^ alters basal gene expression

**Figure 4:**
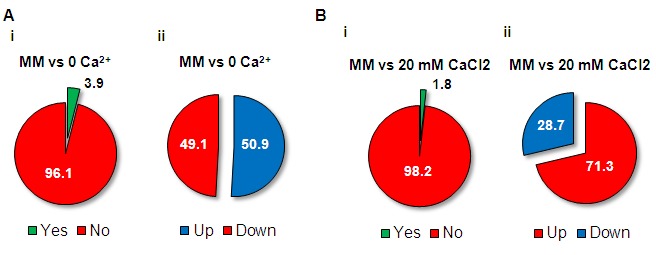
FIGURE 4: Overview of the *N. crassa* transcriptional response to limited or excess Ca^2+^. **(A-B)** The percentages of genes with altered expression (i) and the fraction of induced and repressed genes (ii) were calculated for no Ca^2+^ **(A)** and 20 mM CaCl_2_ medium **(B) **in comparison with standard MM.

**Figure 5:**
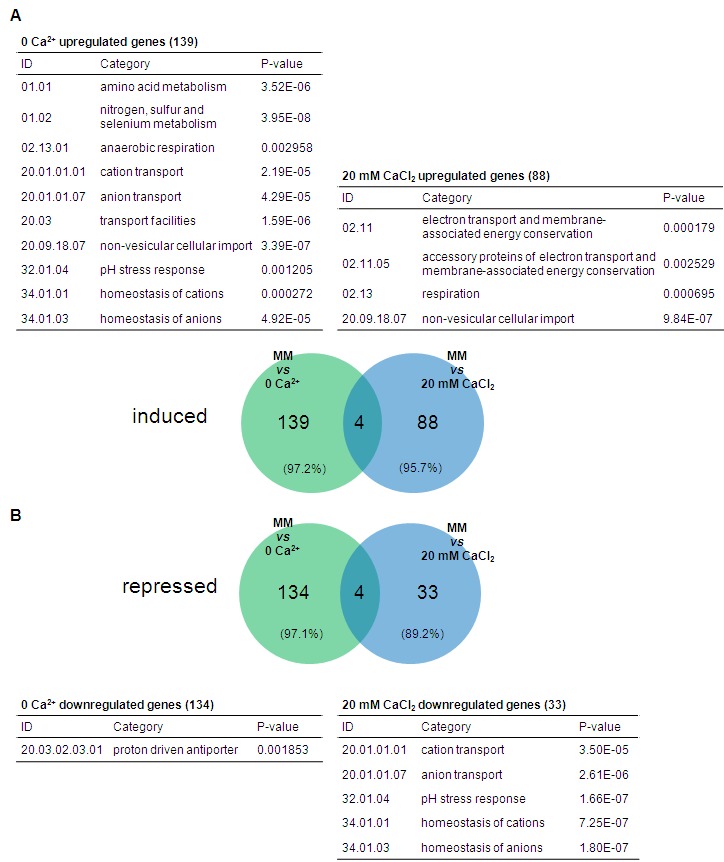
FIGURE 5: Functional enrichment analysis of the transcriptional alterations caused by limited or excess Ca^2+^. **(A-B)** The transcriptional response to the absence or excess of Ca^2+^ was investigated by building Venn diagrams showing the distribution of genes with induced **(A)** or repressed **(B)** expression in no Ca^2+^ and 20 mM CaCl_2_ medium. Lists of enriched categories are included.

The transcriptional response to the lack or excess of Ca^2+^ was analysed by comparing basal gene expression in no Ca^2+^, 20 mM CaCl_2_ and standard MM (File S3). The fraction of genes with altered expression in no Ca^2+^ and 20 mM CaCl_2_ compared with standard MM was 3.9% and 1.8%, respectively (Fig. 4A-B), corresponding to 281 and 129 genes, respectively. While half of the genes altered in no Ca^2+^ were induced and half were repressed, in 20 mM CaCl_2_ most of the altered genes were upregulated (71.3%). Most of the genes were specifically induced or repressed depending on the particular culture medium: 97.2% and 97.1%, respectively, for no Ca^2+^, and 95.7% and 89.2%, respectively, for 20 mM CaCl_2_ (Fig. 5A-B). Not surprisingly, categories related to ‘ion transport’, ‘homeostasis of cations’ and ‘homeostasis of anions’ were enriched in the set of genes induced in the absence of Ca^2+^ (Fig. 5A and File S4), which is perhaps linked to a cellular attempt to recover proper conditions of osmolarity. Consistently, these categories were also significantly enriched among the genes repressed in the presence of 20 mM CaCl_2_ (Fig. 5B). Lack of Ca^2+^ also seems to be associated to a metabolic adaptation of the cells, since there was enrichment in the induction of genes falling on the ‘metabolism’ super-category, like ‘amino acid metabolism’, ‘nitrogen, sulfur and selenium metabolism’ and ‘anaerobic respiration’.

We looked for alterations in the expression of known Ca^2+^ channels, Ca^2+^-ATPases, Ca^2+^-exchangers, Ca^2+^-dependent signaling molecules and other Ca^2+^ binding proteins 18,19,31,32 (Fig. S1 and File S5). NCU11680, NCU04736 and NCU07075, encoding the TRP channel YVC-1, the Ca^2+^-ATPase NCA-2 and the Ca^2+^/H^+^ antiporter CAX, respectively, were repressed in the absence of Ca^2+^. NCU04265, encoding the invertase enzyme (INV) was induced in the absence of Ca^2+^. The induction of INV, an enzyme that hydrolyses extracellular sucrose into glucose and fructose, is a marker of the process of carbon catabolite repression 33,34. NCU08147, encoding the stress-related Ca^2+^-ATPase ENA-2 35 was repressed in the presence of 20 mM CaCl_2_. NCU05046 and NCU07966, encoding ENA-1 and TRM-1, respectively, were induced by no Ca^2+^ and repressed in 20 mM CaCl_2_ medium. These results further suggest an intracellular metabolic remodeling in response to Ca^2+^ availability.

### Two putative novel components of the *N. crassa* Ca^2+^ homeostasis machinery

**Figure 6:**
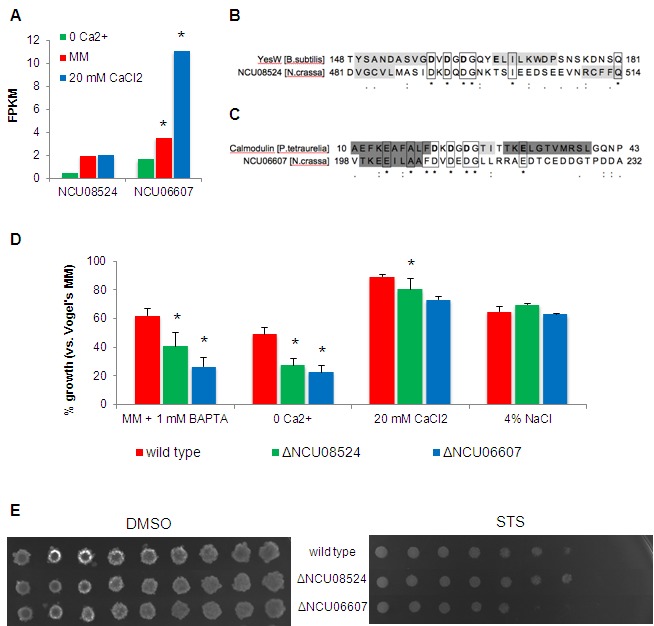
FIGURE 6: Identification of putative novel Ca^2+^-binding proteins. **(A)** Expression levels of NCU08524 and NCU06607 in no Ca^2+^, standard MM and 20 mM CaCl_2_ media. *, p-value <= 0.05. **(B-C)** Sequence alignments of the EF-hand-like domain of NCU08524 and YesW from *B. subtilis* (accession number: O31526; PDB code: 2z8r) **(B),** and NCU06607 and calmodulin from *P. tetraurelia* (accession number: P07463; PDB code: 1exr) **(C)**. Residues binding Ca^2+^ in YesW and calmodulin are in bold; β-strands and α-helices are shaded in light and dark grey, respectively (confirmed by crystallography for YesW 61 and calmodulin 62 and predicted with MEMSAT3 for NCU08524 and NCU06607). **(D)** The percentages of growth of the different strains in the indicated media versus standard MM were determined by measuring absorbance of the respective liquid cultures at 450 nm. *, p-value <= 0.05. **(E)** The spot assay was used to examine the sensitivity of ΔNCU08524 and ΔNCU06607 to 5 μM staurosporine (STS).

We found that 188 genes with unknown function ("hypothetical proteins") had altered expression in no Ca^2+^ or in 20 mM CaCl_2_ medium. We screened the respective protein sequences for the presence of Ca^2+^-binding patterns using CaPS 20 and identified 6 candidates (NCU01697, NCU08524, NCU06116, NCU07582, NCU06607 and NCU03647). We further considered NCU08524 and NCU06607, because the others did not display convincing similarities with known proteins, even around the predicted Ca^2+^-binding motif. NCU08524 was repressed in Ca^2+^-free medium whereas NCU06607 was induced by 20 mM CaCl_2_ (Fig. 6A). Fig. 6B shows the alignment of the predicted Ca^2+^-binding motifs of NCU08524 and NCU06607 with those of proteins containing the Dx[DN]xDG motif 22,23. The NCU08524 motif sequence resembles that of the rhamnogalacturonan lyase YesW from *Bacillus subtilis*. YesW exemplifies β-propeller structures in which the Dx[DN]xDG motif can be embedded in one of the propeller blades, forming a ‘calcium blade’ structure 22. Such a calcium blade was recently described in a fungal lectin from *Psathyrella velutina* 22. NCU08524 is similar to a fucose-specific lectin from *Macrophomina phaseolina* (Table S3) and matches from HHpred suggest that it contains a 6-bladed propeller structure in which β-strands flank the putative Ca^2+^-binding motif. Taking advantage of the similarity between fucose-specific lectins and NCU08524, molecular models were built for the latter in a situation of Ca^2+^ binding (Fig. S2A-C). In proteins containing the Dx[DN]xDG motif, interactions with bound Ca^2+ ^ions are supplemented by the interaction of side chains from at least one downstream acidic residue 22. Therefore, three molecular models were prepared, considering Glu^502^, Glu^503^ or Asp^504^ as the possible additional acidic residue. The models show that NCU08524 can accommodate a Ca^2+^-binding geometry by combining the Dx[DN]xDG motif with any of these downstream residues (Fig. S2A-C).

The predicted Ca^2+^-binding motif of NCU06607 (Fig. 6C) is comparable to the Dx[DN]xDG motif of a calmodulin from *Paramecium tetraurelia* 23. Molecular modeling of NCU06607 was not feasible because the Dx[DN]xDG motif is not located within a folded domain of known structure, but at the beginning of a predicted intrinsically disordered region. Anyway, the Dx[DN]xDG motif is sometimes found in highly disordered regions 36. NCU06607 has a predicted extracellular localization and there is a strong bias in the localization of proteins possessing the Dx[DN]xDG motif towards the cell surface or secretion 22. Plasma membrane localization together with a transmembrane domain was predicted for NCU08524 (Table S3).

The growth of ΔNCU08524 and ΔNCU06607 was compared with the wild type strain under different conditions of Ca^2+^ availability. The growth of both mutants was significantly reduced in no Ca^2+^ medium and in medium containing the Ca^2+^ chelator BAPTA (Fig. 6D). ΔNCU06607 also showed a significant reduction in growth in the presence of 20 mM CaCl_2_ as compared with wild type. The observed differences in growth were not merely due to non-specific osmotic stress, since all strains were similarly affected by the addition of 4% NaCl. ΔNCU06607 was slightly more sensitive to staurosporine than wild type, suggesting that the respective protein may be involved in the response to the drug (Fig. 6E). In summary, the RNA-seq dataset and further analysis revealed two putative novel components of the Ca^2+^-handling machinery in *N. crassa*.

### Several members of the Ca^2+^ homeostasis machinery are involved in the fungal response to staurosporine

**Figure 7:**
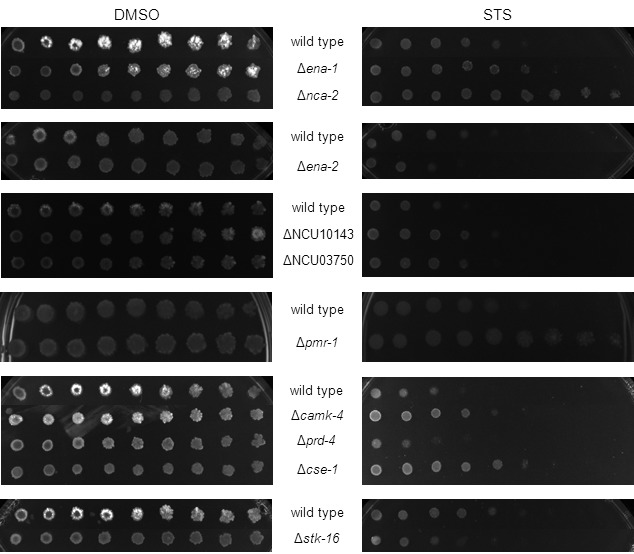
FIGURE 7: Several members of the Ca^2+^ handling machinery are involved in the fungal response to staurosporine. Serial dilutions (from left to right) of conidia from the indicated strains were spotted on GFS medium supplemented with 5 μM staurosporine (STS) and incubated for 3 days.

To further substantiate the crucial role of Ca^2+^ during the *N. crassa* response to staurosporine and to identify mediators of the process, we assayed the drug sensitivity profile of approximately 50 deletion strains encoding proteins involved in Ca^2+^ homeostasis. We showed recently that deletion of genes encoding phospholipase C enzymes, especially PLC-2, results in mutants resistant to staurosporine, while strains lacking the Ca^2+^ channels CCH-1 and MID-1 are hypersensitive 12. Several other deletion strains that lack Ca^2+^-permeable channels, Ca^2+^-ATPases or other Ca^2+^ binding molecules involved in cellular signaling showed a resistance phenotype to staurosporine that was dissimilar from the wild type strain (Fig. 7). A resistance phenotype was particularly strong in deletion mutants for genes encoding the Ca^2+^-ATPases NCA-2 and PMR-1, and deletion of other Ca^2+^-ATPases, namely ENA-1, ENA-2 and NCU10143 also led to increased resistance. The disruption of the signaling molecules calmodulin, CAMK-4, and CSE-1 augmented resistance, whereas strains carrying deletions in genes encoding STK-16 and PRD-4 resulted in increased susceptibility to staurosporine. Table 1 lists strains with altered sensitivity to staurosporine and Table S4 lists all strains tested. The deletion strains for NCU05360, NCU03804, NCU03833, NCU09871, NCU06347, NCU06617, NCU02411 and NCU09265 were not tested due to strain unavailability or their heterokaryotic nature. This data further stresses the importance and complexity of the fungal response to staurosporine, implicating numerous Ca^2+^-related proteins of different function and subcellular localization in the process.

**Table 1:** Strains with Ca2+-related deleted genes that exhibit altered sensitivity to staurosporine when compared with wild type *N. crassa*. SS: much more sensitive; S: slightly more sensitive; RR: much more resistant; R: slightly more resistant.

**Strain**	**Gene name**	**Classification 17,18,19**	**Phenotype**
ΔNCU02762	*cch-1*	Ca^2+^-permeable channel	SS 12
ΔNCU06703	*mid-1*	Ca^2+^-permeable channel	SS 12
ΔNCU04736	*nca-2*	Ca^2+^-ATPase	RR
ΔNCU05046	*ena-1*	Ca^2+^-ATPase	R
ΔNCU08147	*ena-2*;* ph-7*	Ca^2+^-ATPase	S
ΔNCU10143		Ca^2+^-ATPase	R
ΔNCU03292	*pmr-1*	Ca^2+^-ATPase	RR
ΔNCU06245	*plc-1*	Phospholipase C (δ-type)	R 12
ΔNCU01266	*plc-2*	Phospholipase C (δ-type)	RR 12
ΔNCU09655	*plc-3*	Phospholipase C (δ-type)	R 12
ΔNCU03750		Calmodulin	R
ΔNCU04379	*cse-1*	Ca^2+^ and/or CaM binding protein	R
ΔNCU09212	*camk-4*	Ca^2+^ and/or CaM binding protein	R
ΔNCU00914	*stk-16*	Ca^2+^ and/or CaM binding protein	S
ΔNCU02814	*prd-4*	Ca^2+^ and/or CaM binding protein	S

## DISCUSSION

Here we show that controlling the amount of Ca^2+^ available in the extracellular milieu is enough for a robust modulation of the effects of staurosporine in *N. crassa*. Cell death is strongly enhanced by Ca^2+^ limitation whereas it is partially suppressed by a ~30-fold excess of external Ca^2+^. A similar behavior was observed in *S. cerevisiae* cells treated with miconazole or terbinafine, inhibitors of ergosterol biosynthesis 37, suggesting common stress-responsive pathways in the two fungi, even when the stimuli have apparently distinct targets. Supplementation of the medium with high Ca^2+^ concentrations (20 mM CaCl_2_, as used here) protected *N. crassa *cells from toxicity induced by PAF, an antifungal protein from *Penicillium chrysogenum* that also disturbs intracellular Ca^2+^ homeostasis 10. Our results substantiate the view that Ca^2+^ is an important player during cell death.

The effects of staurosporine in media containing different amounts of Ca^2+^ are remarkably paralleled by distinct cytosolic Ca^2+^ dynamics, in line with our previous findings that extracellular Ca^2+^ uptake is required for the development of the cytosolic Ca^2+^ signature induced by staurosporine 12. We have recently demonstrated that during the staurosporine-induced Ca^2+^ signature, peaks "A" and "B" precede cell death, suggesting that they represent signaling events. In the presence of 20 mM CaCl_2_ the peak "A" is enhanced, whereas in medium lacking Ca^2+^ peaks "A" and "B" of the staurosporine-induced cytosolic Ca^2+^ response are abolished. Thus, intracellular Ca^2+^ signaling that is triggered by staurosporine is markedly modulated by the concentration of Ca^2+^ in the external medium and might have a role in determining cell survival. However, the intracellular response to staurosporine is very complex and direct conclusions on the susceptibility of the cells based solely on the cytosolic Ca^2+^ profile, although tempting, are not possible. For instance, a Δ*plc-2* mutant shows increased resistance to the drug despite that its cytosolic Ca^2+^ response to staurosporine is nearly obliterated 12. Thus, it seems that events downstream of the cytosolic changes in Ca^2+^ levels ultimately define cell fate in response to staurosporine.

The screening for staurosporine sensitivity of Ca^2+^-handling mutant strains revealed a number of proteins likely involved in the cell death response to the drug. Further studies are needed to contextualize them functionally in the mechanism of action of the drug. NCA-2 is a Ca^2+^-ATPase which pumps excess of cytosolic Ca^2+^ to the vacuoles or extracellular space 38,39, and the respective knockout cells are very resistant to staurosporine. The knockout strain for PMR-1, a Golgi-localized Ca^2+^-ATPase 40, is also very resistant to staurosporine and yeast Δ*PMR1* knockout cells accumulate much less Ca^2+^ in the ER 41. Lack of these functions in Δ*nca-2* and Δ*pmr-1* cells is likely related to the phenotype of the strains upon treatment with staurosporine, as both the ER and the vacuoles play an important role during the fungal response to staurosporine 12. It is plausible that the lack of NCA-2 or PMR-1 results in an increased accumulation of Ca^2+^ in the cytosol and this is consistent with the observations that high levels of Ca^2+^ (below a toxicity threshold) protect against the effects of staurosporine. The lack of NCA-2 has been recently associated with increased sensitivity to UV exposure, further implicating the molecule in cell survival 42. Other Ca^2+^-related deletion strains present altered resistance to staurosporine, including Δ*cse-1*, Δ*camk-4* and Δ*stk-16*. Knockout of *cse-1* has been shown to lead to impaired germling communication and fusion 43, as well as increased sensitivity to UV irradiation and Ca^2+^ 44. A previous study has shown that disruption of *camk-4 *results in benomyl resistance, while disruption of *stk-16* leads to increased sensitivity to fludioxonil, sodium chloride and benomyl 45.

The *N. crassa *transcriptional response to staurosporine is robustly reduced in the presence of 20 mM CaCl_2_ when compared with standard MM, whereas limited extracellular Ca^2+^ causes alterations in an increased number of genes. Thus, different concentrations of extracellular Ca^2+^ not only lead to distinct intracellular Ca^2+^ dynamics, ROS accumulation and cell death levels, but also seem to be coupled to unique transcriptional profiles. It appears that, in the absence of Ca^2+^, cells treated with staurosporine turn off a diverse array of biological processes. We discovered several enriched functional categories in the set of no Ca^2+^ medium-specific repressed genes, including the repression of genes involved in cell cycle, signal transduction, cell growth, anti-apoptosis, cellular polarization, protein fate and the unfolded protein response. It is possible that the decreased activity of various intracellular pathways explains the inability of cells to cope with the staurosporine insult and the consequent increased cell death.

*N. crassa* clearly translates changes in extracellular Ca^2+^ into a transcriptional response, since alterations in gene expression are caused alone by extracellular Ca^2+^ limitation or overload. This likely results from the homeostatic adaptation to the abnormal Ca^2+^ environment, i.e., the observed differences in gene expression might be triggered after the uptake or release of Ca^2+^ to balance the intracellular levels of the ion. Alternatively, these alterations may be secondary to the activity of a surface-localized Ca^2+^ sensor, although a sequence homologue of the extracellular Ca^2+^-sensing receptor of animals and plants 46,47 has not been found so far in *N. crassa*. Given the predicted cell surface localization of the two putative novel Ca^2+^-binding proteins, NCU08524 and NCU06607, and the growth phenotype of the respective deletion strains under Ca^2+^ stress, it is tempting to speculate that they could play a role in Ca^2+^ sensing. The chemotropic interaction during conidial anastomosis tubes during cell fusion is Ca^2+^-dependent 43 and both NCU08524 and NCU06607 genes have been recently shown to be under the control of the PP-1 transcription factor, a regulator of cell fusion 48,49.

In summary, we show that the effects of the antifungal 13,14,15,16,50 and anticancer 51 agent staurosporine are robustly modulated by the accessibility to Ca^2+^ in the culture medium. The amount of extracellular Ca^2+^ affects staurosporine-triggered intracellular events like Ca^2+^ signaling, the production of ROS and cell death. Our results indicate that these differences are linked to the stimulation of unique transcriptional programs and further highlight the importance of Ca^2+^ during fungal cell death. In addition, our RNA-seq dataset will be a useful resource for investigations on the role of Ca^2+^ on different aspects of fungal biology.

## MATERIALS AND METHODS

### Strains, culture media and chemicals

Wild type 74-OR23-1VA strain and deletion mutants were obtained from the Fungal Genetics Stock Center 52 and handled with standard procedures 28. Minimal medium refers to Vogel’s medium containing 1.5% (w/v) sucrose 27 plus 1.5% (w/v) agar to obtain solid medium. The concentration of KH_2_PO_4_ in the 50x Vogel’s stock solution was limited to 10 mM to avoid precipitation with supplemental calcium 10. The following chemicals were used: staurosporine (from LC Laboratories), dimethyl sulfoxide (DMSO), A23187, calcium chloride and sodium chloride (Sigma-Aldrich), phytosphingosine (Avanti Polar Lipids), 1,2-bis(ortho-aminophenoxy)ethane-N,N,N’,N’-tetrasodium (BAPTA) (Merck Biochemicals).

### Growth assays

Hyphal growth in solid medium at 26°C was obtained by measuring colony elongation after the inoculation of 20 μl containing 5x10^4^ conidia on the centre of large Petri dishes (ϕ 14.2 cm). Growth in liquid medium was examined by incubating 1x10^4^ conidia/ml at 26°C, 100 rpm, under constant light in 96-well plates (200 μl/well) and following absorbance at 450 nm 53 during 24 hours. The % growth versus the respective control and the growth rate were calculated for each condition. For the spot assays, 3-fold serial dilutions up to 1x10^4^ cells/ml were prepared for each strain. 5 μl from each dilution were spotted on plates containing glucose-fructose-sorbose (GFS) 28 plus the relevant chemicals and incubated for 3 days at 26°C.

### Flow cytometry measurements of cell death and reactive oxygen species (ROS)

For each experiment and strain, a control without staining was prepared to define autofluorescence. Samples were read in a BD FACS Calibur and data analyzed with FlowJo (Tree Star). The levels of cell death were determined by staining cells with YOPRO-1 (Life Technologies) or propidium iodide (PI; Sigma-Aldrich). An inoculum of 10^6^ conidia/ml in the appropriate medium was incubated for 4 hours at 26°C (140 rpm, constant light). Staurosporine (20 μM) was added and growth resumed for further 2 hours. The cells were harvested by centrifugation, washed twice with PBS and incubated either with 0.1 μM YOPRO-1 (samples were kept 20 min on ice before reading) or 2 μg/ml PI (samples were immediately ready for cytometry). The production of ROS was measured using the fluorescent probe dihydrorhodamine 123 (Sigma-Aldrich). After growing 10^6^ conidia/ml for 4 hours in the indicated medium at 26°C, 20 μg/ml dihydrorhodamine 123 and staurosporine were added for further 30 minutes. Samples were harvested by centrifugation and washed twice with PBS before being resuspended in PBS and read in the cytometer.

### Cytosolic Ca^2+^ measurements

A bioluminescent method based on the reaction of cytosolic Ca^2+^ with the genetic probe aequorin in wild type cells was employed as previously described 12. Briefly, 100 μl of conidia at a concentration of 2x10^6^ cells/ml, 5 μM coelenterazine (Santa Cruz Biotechnology) and minimal medium were added to white opaque 96-well plates and incubated for 6 hours at 26°C, in the dark, without agitation. Luminescence (in RLU, relative light units) was captured over time on a Bio-Tek Synergy HT microplate reader. Maximum levels of luminescence, determined in extra wells by measuring luminescence during 3 mins after the injection of 100 μl of 3M CaCl_2_ in 20% ethanol, were used to normalize each experiment (cytosolic Ca^2+^ levels (arbitrary units) = experimental RLU values / total emitted luminescence). Quantification was performed by summing the normalized values and data are expressed as mean ± SEM. The values from control DMSO-treated samples were subtracted from staurosporine-treated samples to obtain the "staurosporine-induced amplitude of response".

### Statistical and protein sequence analyses

The non-parametric Mann-Whitney test was used for comparisons between two groups using SPSS 20 (SPSS Inc.) and p-values <= 0.05 were considered statistically significant. ClustalW2 54 was used to align protein sequences and PSI-BLAST 55 was used for iterative searches. Secondary structure was predicted with MEMSAT3 56, conserved domains with InterProScan 57, transmembrane domains with TMHMM 2.0 58, subcellular localization with WoLF PSORT 59 and Ca^2+^-binding motifs with CaPS 20.

### High-throughput RNA sequencing (RNA-seq)

Conidial suspensions of 1x10^6^ cells/ml were incubated in culture medium with the indicated concentration of Ca^2+^ for 6 hours (26°C, 140 rpm, constant light), staurosporine (or DMSO alone) was added and growth resumed for 1 more hour. Cells were harvested and immediately frozen in liquid nitrogen. RNA isolation, mRNA purification and fragmentation and cDNA synthesis was performed as previously described 15. The cDNA libraries were generated using an Illumina TruSeq kit and sequenced in an Illumina HiSeq2000 (single reads of 50 bp were obtained). Sequencing data was handled with Tophat, Cufflinks and Cuffdiff and expression levels are presented as Fragments Per Kilobase of transcript per Million mapped reads (FPKM). Functional enrichment of sets of genes was assessed with FunCat 60. The resulting dataset is available at the NCBI GEO database (http://www.ncbi.nlm.nih.gov/geo; series record: GSE53013).

## SUPPLEMENTAL MATERIAL

Click here for supplemental data file.

All supplemental data for this article are also available online at http://microbialcell.com/researcharticles/extracellular-calcium-triggers-unique-transcriptional-programs-and-modulates-staurosporine-induced-cell-death-in-Neurospora-crassa/.
