# Supplementary material for: Extracellular calcium triggers unique transcriptional programs and modulates staurosporine-induced cell death in Neurospora crassa
Source: Microb Cell. 2014 Aug 9;1(9):289–302. doi: 10.15698/mic2014.09.165 (PMC5349132; doi:10.15698/mic2014.09.165)
Supplement: Supplementary file 1 [file mic-01-289-s01.pdf]

Extracellular calcium triggers unique transcriptional programs and modulates staurosporine-induced cell death in *Neurospora crassa*

Supplemental data

Figure S1 - Expression levels of selected genes encoding components of the Ca^{2+}-machinery in the different culture media: NCU11680/*yvc-1* (A), NCU04736/*nca-2* (B), NCU07075/*cax* (C), NCU04265/*inv* (D), NCU08147/*ena-2* (E), NCU05046/*ena-1* (F) and NCU07966/*trm-1* (G).

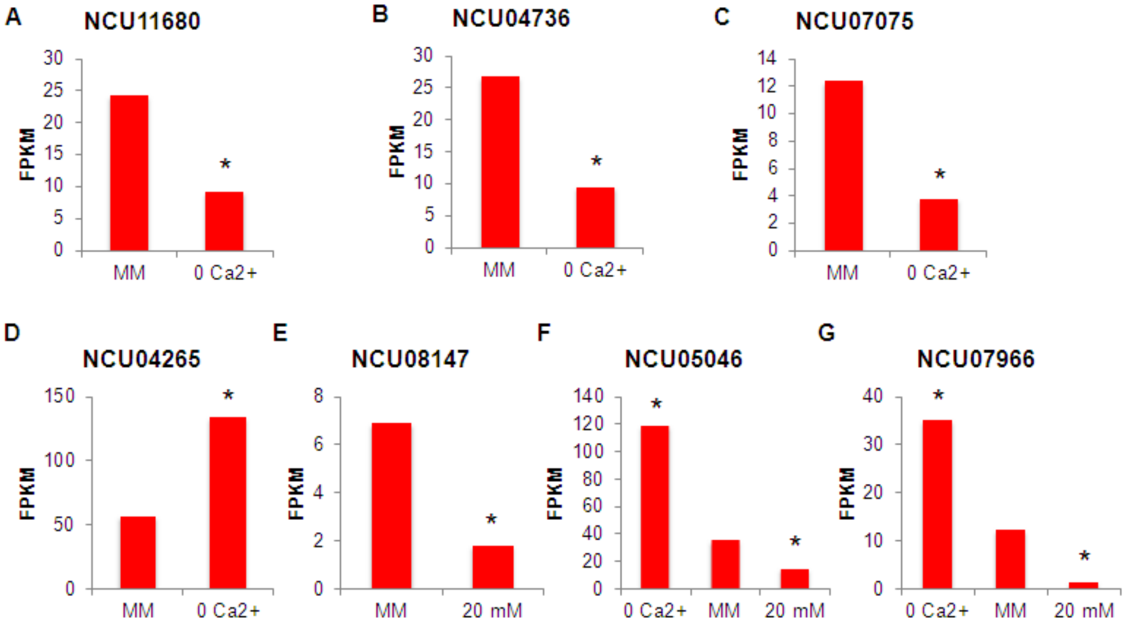

Figure S2 - Molecular models of NCU08524. Glu^{502}, Glu^{503} or Asp^{504} was used as the additional acidic Ca^{2+} binding residue downstream of the Dx[DN]xDG motif (A, B and C, respectively). Templates to model the candidate novel Ca^{2+}-binding proteins were sought using HHpred [1]. This pinpointed *Aspergillus fumigatus* and *Aleuria aurantia* fucose-binding lectins (PDB codes 4ah5 and 1ofz, respectively) as suitable for modeling NCU08524. No templates were available for modeling NCU06607. NCU08524 models were constructed with MODELLER [2] using the target-template alignments from HHpred and additionally employing the Dx[DN]xDG motif from the second calcium blade of *Psathyrella velutina* lectin (PDB code 2bwr) to specify appropriate local geometry for the putative Ca^{2+}-binding motif of NCU08524. Alternative additional calcium ligating residues - Glu^{502}, Glu^{503} or Asp^{504} - were explored, and Ca^{2+}-protein distance restraints of 2.4 Å (side chain) or 2.3 Å (main chain) applied to enforce the canonical metal ligation pattern of Dx[DN]xDG motifs. The models are shown in PyMOL (http://www.pymol.org/) as cartoons coloured by secondary structure (magenta: β-strands; cyan: α-helices). Bound Ca^{2+} is shown as a grey sphere and ligating residues are shown as sticks in yellow (Asp residues of the Dx[DN]xDG motif), blue (the motif's Gly residue) or green (the additional acidic residue).

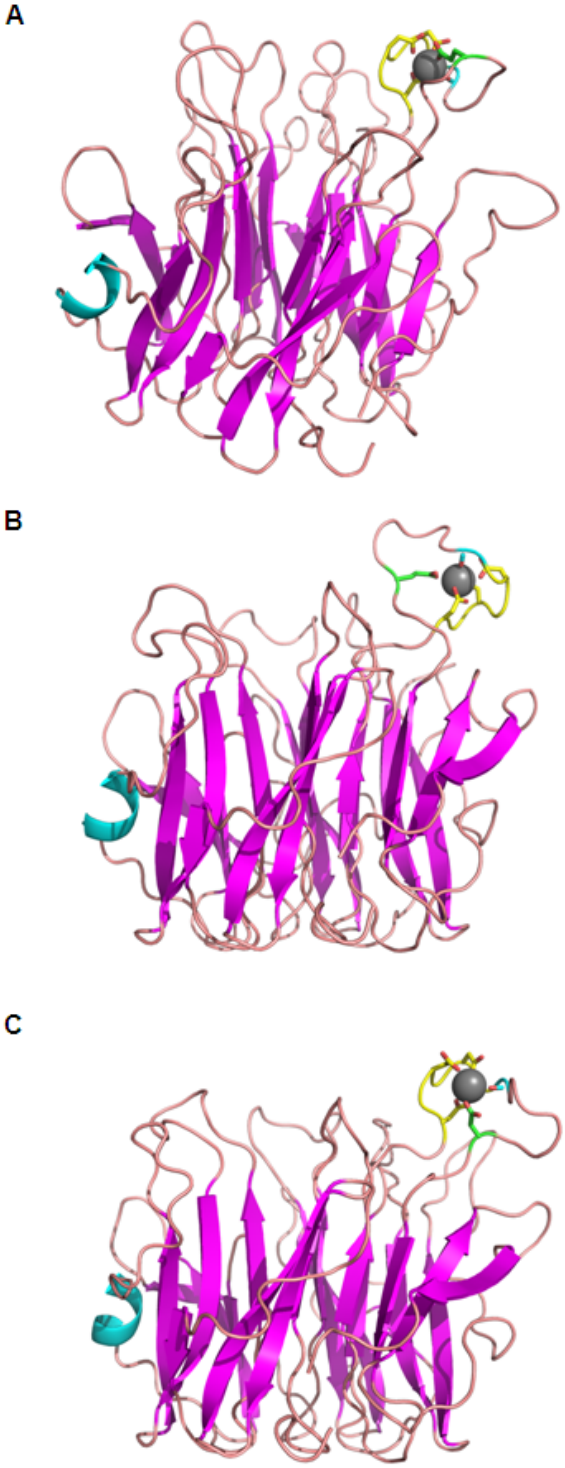

A
B
C

Table S1 - Functional enrichment analysis (FunCat) of *N. crassa* genes induced by staurosporine in the specific culture media.

ID	Category	P-value
Standard MM		
01.06	lipid, fatty acid and isoprenoid metabolism	5.03E-06
14.01	protein folding and stabilization	1.05E-04
14.07	protein modification	7.33E-06
14.07.02	modification with sugar residues (eg, glycosylation, deglycosylation)	8.58E-07
20.01.03	C-compound and carbohydrate transport	8.20E-04
20.01.13	lipid/fatty acid transport	1.58E-04
20.09	transport routes	2.15E-05
20.09.07	vesicular transport (Golgi network, etc)	2.81E-07
20.09.16	cellular export and secretion	9.91E-05
0 Ca^{2+}		
01.20.01.09	metabolism of aminoglycoside antibiotics	0.0065
01.25	extracellular metabolism	0.0080
01.25.11	extracellular aminosaccharide degradation	0.0065
20 mM $CaCl_2$		
11.04	RNA processing	3.94E-05
11.04.01	rRNA processing	1.84E-04

Table S2 - Functional enrichment analysis (FunCat) of *N. crassa* genes repressed by staurosporine in the specific culture media.

ID	Category	P-value
Standard MM		
02.11	electron transport and membrane-associated energy conservation	1.05E-08
02.13	respiration	2.46E-10
02.13.03	aerobic respiration	3.27E-09
02.45	energy conversion and regeneration	1.54E-05
02.45.15	energy generation (eg, ATP synthase)	6.43E-09
12.01	ribosome biogenesis	1.75E-34
12.01.01	ribosomal proteins	3.74E-40
12.04	translation	4.61E-28
16.01	protein binding	3.14E-11
16.03.03	RNA binding	6.12E-05
20.01.15	electron transport	5.26E-05
20.09.04	mitochondrial transport	0.0002
34.01.01.03	homeostasis of protons	9.98E-05
0 Ca^{2+}		
01.01	amino acid metabolism	1.19E-06
01.01.06	metabolism of the aspartate family	2.75E-05
01.03	nucleotide/nucleoside/nucleobase metabolism	1.63E-06
01.03.01	purine nucleotide/nucleoside/nucleobase metabolism	6.05E-05
01.03.16.01	RNA degradation	0.0002
01.04	phosphate metabolism	4.61E-07
01.05.02.04	sugar, glucoside, polyol and carboxylate anabolism	0.0032
01.05.05.04	C- compound anabolism	0.0061
01.05.09.04	aminosaccharide anabolism	0.0056
01.06	lipid, fatty acid and isoprenoid metabolism	2.16E-05
01.06.06.11	tetracyclic and pentacyclic triterpenes (cholesterin, steroids and hopanoids) metabolism	7.56E-06
02.01	glycolysis and gluconeogenesis	0.0007

02.07	pentose-phosphate pathway	0.0011
10	cell cycle and DNA processing	2.35E-07
11.02	RNA synthesis	1.74E-08
11.02.03	mRNA synthesis	7.29E-07
11.04	RNA processing	3.07E-07
11.04.03	mRNA processing (splicing, '-, '-end processing)	3.84E-05
14	protein fate (folding, modification, destination)	8.30E-06
18	regulation of metabolism and protein function	4.69E-05
20.01.10	protein transport	9.40E-05
20.03	transport facilities	5.89E-05
20.09	transport routes	2.83E-11
30	cellular communication/signal transduction mechanism	6.17E-05
32.01.07	unfolded protein response (eg, ER quality control)	1.34E-05
34.11	cellular sensing and response to external stimulus	1.39E-06
40.01	cell growth / morphogenesis	8.94E-06
40.10.02.01	anti-apoptosis	0.0026
42.01	cell wall	8.82E-06
42.04	cytoskeleton/structural proteins	3.62E-05
43.01.03.05	budding, cell polarity and filament formation	5.28E-05
20 mM $CaCl_2$		
01.01	amino acid metabolism	1.20E-05
01.01.03	assimilation of ammonia, metabolism of the glutamate group	0.0003
01.06	lipid, fatty acid and isoprenoid metabolism	0.0006
01.07	metabolism of vitamins, cofactors, and prosthetic groups	0.0043
01.20.37.01	metabolism of thioredoxin, glutaredoxin, glutathione	0.0010
02.11	electron transport and membrane-associated energy conservation	9.03E-05
20.01.15	electron transport	0.0001

Table S3 - Protein sequence features of the two putative novel Ca^{2+}-binding proteins NCU08524 and NCU06607.

**Protein**	**Ca^{2+}-binding motif [a]**	**CD [b]**	**TMD [c]**	**SL [d]**	**Database search results [e]**
NCU08524	491-DKDQDGNKTSIEE-503	Fucose-specific lectin	1	PM: 18; Nu: 4; Mi: 2; Cy: 1	Fungal fucose-specific lectin (*M. phaseolina*) [7E-07; 2]<br>Serine/threonine kinase PKN13 (*C. apiculatus*) [1E-03; 2]
NCU06607	208-DVDEDGLLRRAED -220	No hits	0	Ex: 25	Hemolysin-type calcium-binding protein (*T. xiamenensis*) [3E-08; 2];<br>FecR (*C. stagnale*) [2E-07; 2];<br>von Willebrand factor A (*H. salifodinae*) [3E-07; 2];<br>Cell surface antigen Sca13 (*R. bellii*) [6E-07; 2]<br>Halomucin (*H. walsbyi*) [1E-06; 2]<br>Antigen Cs44 (*C. sinensis*) [1E-04; 2]

[a] Ca^{2+}-binding motifs were predicted with CaPS [3]. [b] Conserved domains were predicted with InterProScan [4]. [c] The number of transmembrane domains was predicted with TMHMM 2.0 [5]. [d] Subcellular localization was predicted with WoLF PSORT [6]; PM: plasma membrane; ER: endoplasmic reticulum; Nu: nuclear; Mi: mitochondria; Cy: cytosol; Ex: extracellular; Go: Golgi apparatus; the value in front of each prediction corresponds to the score (the higher the score, the more reliable is the prediction). [e] Homology was predicted with PSI-BLAST [7]; the value in front of each prediction (between square brackets) corresponds to the E-value and the PSI-BLAST iteration number.

Table S4 - List of deletion strains tested for resistance to staurosporine and the respective phenotype.

**Deleted gene**	**Classification [8-10]**	**Phenotype (*versus* wild type)**
NCU02762	Ca^{2+} permeable channel	Much more sensitive [11]
NCU06703	Ca^{2+} permeable channel	Much more sensitive [11]
NCU16725	Ca^{2+} permeable channel	= [11]
NCU02219	Ca^{2+} permeable channel	Slightly more sensitive [11]
NCU03305	Ca^{2+}-ATPase	=
NCU04736	Ca^{2+}-ATPase	Much more resistant
NCU05154	Ca^{2+}-ATPase	=
NCU03292	Ca^{2+}-ATPase	Much more resistant
NCU08147	Ca^{2+}-ATPase	Slightly more sensitive
NCU04898	Ca^{2+}-ATPase	=
NCU03818	Ca^{2+}-ATPase	=
NCU05046	Ca^{2+}-ATPase	Slightly more resistant
NCU07966	Cation ATPase	=
NCU10143	Cation ATPase	Slightly more resistant
NCU07075	Ca^{2+}/H^{+} exchanger	=
NCU00916	Ca^{2+}/H^{+} exchanger	=
NCU00795	Ca^{2+}/H^{+} exchanger	=
NCU06366	Ca^{2+}/H^{+} exchanger	=
NCU07711	Ca^{2+}/H^{+} exchanger	=
NCU02826	Ca^{2+}/Na^{+} exchanger	=
NCU08490	Ca^{2+}/Na^{+} exchanger	=
NCU01266	Phospholipase C	Much more resistant [11]
NCU06245	Phospholipase C	Slightly more resistant [11]
NCU09655	Phospholipase C	Slightly more resistant [11]
NCU02175	Phospholipase C	= [11]
NCU04120	Calmodulin	=
NCU03750	Calmodulin	Slightly more resistant
NCU04421	Calpactin I heavy chain	=
NCU02115	Ca^{2+} and/or CaM binding protein	=
NCU01564	Ca^{2+} and/or CaM binding protein	=
NCU06948	Ca^{2+} and/or CaM binding protein	=
NCU04379	Ca^{2+} and/or CaM binding protein	Slightly more resistant
NCU02738	Ca^{2+} and/or CaM binding protein	=
NCU01241	Ca^{2+} and/or CaM binding protein	=
NCU02283	Ca^{2+} and/or CaM binding protein	=
NCU09123	Ca^{2+} and/or CaM binding protein	=

NCU02814	Ca^{2+} and/or CaM binding protein	Slightly more sensitive
NCU09212	Ca^{2+} and/or CaM binding protein	Slightly more resistant
NCU06650	Ca^{2+} and/or CaM binding protein	=
NCU06177	Ca^{2+} and/or CaM binding protein	=
NCU04265	Ca^{2+} and/or CaM binding protein	=
NCU00914	Ca^{2+} and/or CaM binding protein	Slightly more sensitive

File S1 - Staurosporine-induced transcriptional response of *N. crassa* (RNA-seq dataset).

File S2 - Functional enrichment analysis (FunCat) of Ca^{2+}-specific staurosporine-induced and -repressed genes.

File S3 - Ca^{2+}-induced transcriptional response of *N. crassa* (RNA-seq dataset).

File S4 - Functional enrichment analysis (FunCat) of 0 Ca^{2+}- and 20 mM $CaCl_2$-specific induced and repressed genes.

File S5 - Expression levels of members of the Ca^{2+}-handling machinery in 0 Ca^{2+} and 20 mM $CaCl_2$ in comparison with standard MM.

Supplemental References

1. Soding J, Biegert A, Lupas AN (**2005**). The HHpred interactive server for protein homology detection and structure prediction. **Nucleic Acids Res** 33(Web Server issue): W244-248. doi:10.1093/nar/gki40.

2. Sali A, Blundell TL (**1993**). Comparative protein modelling by satisfaction of spatial restraints. **J Mol Biol** 234(3): 779-815. doi: 10.1006/jmbi.1993.1626.

3. Zhou Y, Yang W, Kirberger M, Lee HW, Ayalasomayajula G, Yang JJ (**2006**). Prediction of EF-hand calcium-binding proteins and analysis of bacterial EF-hand proteins. **Proteins** 65(3): 643-655. doi:10.1002/prot.21139.

4. Quevillon E, Silventoinen V, Pillai S, Harte N, Mulder N, Apweiler R, Lopez R (**2005**). InterProScan: protein domains identifier. **Nucleic Acids Res** 33(Web Server issue): W116-120. doi:10.1093/nar/gki442.

5. Krogh A, Larsson B, von Heijne G, Sonnhammer EL (**2001**). Predicting transmembrane protein topology with a hidden Markov model: application to complete genomes. **J Mol Biol** 305(3): 567-580. doi:10.1006/jmbi.2000.4315.

6. Horton P, Park KJ, Obayashi T, Fujita N, Harada H, Adams-Collier CJ, Nakai K (**2007**). WoLF PSORT: protein localization predictor. **Nucleic Acids Res** 35(Web Server issue): W585-587. doi:10.1093/nar/gkm259.

7. Altschul SF, Madden TL, Schaffer AA, Zhang J, Zhang Z, Miller W, Lipman DJ (**1997**). Gapped BLAST and PSI-BLAST: a new generation of protein database search programs. **Nucleic Acids Res** 25(17): 3389-3402. doi:10.1093/nar/25.17.3389.

8. Borkovich KA, Alex LA, Yarden O, Freitag M, Turner GE, Read ND, Seiler S, Bell-Pedersen D, Paietta J, Plesofsky N, Plamann M, Goodrich-Tanrikulu M, Schulte U, Mannhaupt G, Nargang FE, Radford A, Selitrennikoff C, Galagan JE, Dunlap JC, Loros JJ, Catcheside D, Inoue H, Aramayo R, Polymenis M, Selker EU, Sachs MS, Marzluf GA, Paulsen I, Davis R, Ebbole DJ, et al. (**2004**). Lessons from the genome sequence of *Neurospora crassa*: tracing the path from genomic blueprint to multicellular organism. **Microbiol Mol Biol Rev** 68(1): 1-108. doi:10.1128/MMBR.68.1.1-108.2004.

9. Galagan JE, Calvo SE, Borkovich KA, Selker EU, Read ND, Jaffe D, FitzHugh W, Ma LJ, Smirnov S, Purcell S, Rehman B, Elkins T, Engels R, Wang S, Nielsen CB, Butler J, Endrizzi M, Qui D, Ianakiev P, Bell-Pedersen D, Nelson MA, Werner-Washburne M, Selitrennikoff CP, Kinsey JA, Braun EL, Zelter A, Schulte U, Kothe GO, Jedd G, Mewes W, et al. (**2003**). The genome sequence of the filamentous fungus *Neurospora crassa*. **Nature** 422(6934): 859-868. doi:10.1038/nature01554.

10. Zelter A, Bencina M, Bowman BJ, Yarden O, Read ND (**2004**). A comparative genomic analysis of the calcium signaling machinery in *Neurospora crassa*, *Magnaporthe grisea*, and *Saccharomyces cerevisiae*. **Fungal Genet Biol** 41(9): 827-841. doi:10.1016/j.fgb.2004.05.001.

11. Gonçalves AP, Cordeiro JM, Monteiro J, Muñoz A, Correia-de-Sá P, Read ND, Videira A (**2014**). Activation of a TRP-like channel and intracellular calcium dynamics during phospholipase C-mediated cell death. **J Cell Sci**. (In press) doi:10.1242/jcs.152058.
